# Odontological Society of New York

**Published:** 1885-02

**Authors:** 


### ODONTOLOGICAL SOCIETY OF NEW YORK.

The January meeting of this society was held at the residence of Dr. W. E. Hoag.

Dr. Raymond was introduced to the society, and read an exceedingly interesting paper on "Hydrochlorate of Cocaine," citing five cases in his own practice in which the anæsthetic had been used. The instances in which it was applied to the tooth substance did not give very satisfactory results, even where the cavities had been thoroughly dried, and the hot air syringe used. He believes the best effects are obtained by a hypodermic injection of the drug at a point near the nerve trunk. In this manner he has produced anæsthesia readily, and had prepared and filled otherwise exceedingly sensitive teeth with no after effects other than slight soreness for a short time, complete recovery from the anæsthesia taking place in from twenty-five to thirty-five minutes. He advised that fresh solutions be made from the crystals dissolved in distilled water. Care should be taken not to introduce air into the tissues with the hypodermic syringe. To prevent this the syringe should be fully charged with the fluid, thus excluding all air. Sufficient of the fluid should then be forced out to leave only the exact quantity required for use. To prevent any pain from the introduction of the needle, Dr. Raymond saturates a pellet of cotton with the cocaine, and touches the spot a minute or two before the puncture.

*Dr. Perry*—Stated that a meeting of a few gentlemen, both Dr. Bogue and himself had been injected with the cocaine; the introduction of the needle being near the inferior dental nerve at the mental foramen, causing complete loss of feeling in the lower lip of that side, and extending somewhat to the tongue and the side of the face. The next morning he experienced a feeling of soreness in that locality, and an uncomfortable tingling feeling had been noted for a day or two since, causing him to unconsciously put his hand to that side of the face. He made this statement hesitatingly, as the peculiar sensation might be due to the fact that he had been exceedingly busy and worried for a day or two, and had slept very little at night. He thought, as Dr. Bogue had not been similarly affected, it might possibly be nervousness in his case.

*Dr. Bogue*—Stated that he felt no after effects from the hypodermic administration of the cocaine, except, perhaps, a slight soreness, which soon passed off. With reference to the use of the hot air syringe, Dr. Bogue questioned if Dr. Raymond was able to get a continuous hot air current, and thought the pain in the tooth might have been from sudden changes of temperature, as Dr. Bogue had never seen but one syringe capable of giving a steady blast of hot air, and that one was invented by Dr. Brasseur, of Paris.

*Dr. Woodward*—Asked if any cases of nausea had been noticed in using the new anæsthetic.

*Dr. Raymond*—Had one case, that of a young miss, but the nausea was very slight, and soon passed off.

*President Jarvie*—Stated that he would be pleased to hear remarks on Dr. Beers' paper, read at the last meeting.

*Dr. Dodge*—Said that Dr. Beers had in his paper simply made engaging and fascinating statements concerning an old theory, but he thought the society was indebted to him for bringing up the subject, which was an important and interesting one.

*Prof. Mayr*, (of Springfield)—Entertained the society with a lecture on "The Products of Putrefaction and Fermentation in the Mouth," in which some very interesting chemical processes were described. He did not think that acid conditions in the mouth had as much to do with caries in teeth as was generally supposed, but considered that antacid washes and tooth powders, though harmless, did little good. He advised a thorough cleansing of the teeth and mouth, and the employment of antiseptics, such as carbolic acid, in dilute washes. To substantiate his theory, he stated that of the peasants of Germany many ate sauer-kraut for breakfast, sauer-kraut for dinner, and sauer-kraut for supper, without so much as picking the particles from between the teeth; and still their dentures would compare favorably with those of young ladies of this country. It was his opinion that good or poor qualities of teeth were an inheritance; that if a young man had good front teeth and the molars all broken down with decay, it was a certain indication that his father's teeth were similarly affected.

*Dr Clowes*—Thought much of the pitted and poor condition of teeth should be laid at the physician's door, as they were constantly prescribing strong acids and iron preparations, which, though taken through a glass tube, would follow the mucous membrane back to the mouth and affect the teeth.

*Dr. Dwinelle*—Thought Prof. Mayr's lecture full of interesting chemical mysteries, but that the profession ought not to accept new theories too readily, when years of constant practical experience had made positive certain antagonistic facts. He had not the slightest doubt that acids dissolved out the lime salts, and thus caused the breaking down of tooth structure.

